# National Health and Modern Problems

**Published:** 1920-07-17

**Authors:** 


*To the Editor of* The Hospital.

### National Health and Modern Problems.

*To the Editor of* The Hospital.

Sir,—The professional man, as becomes his responsible office, deems it his duty to point out to the public generally how much depends on individual effort to bring about the physical betterment of the race. As a mere man in the street, while heartily concurring in this attitude, I would like to make terse comment on "the system" with which the worker contends.

It seems very hard that so many men must, of sheer necessity, accept lifelong employment in occupations which entail chronic constipation (look at the pills sold), excessive physical strain, or practically no exercise at all, a cutting off from sunshine and fresh air, or, worse still, rotting in some form or other from chemicals. The statistics of occupational ailment and disease are appalling. Indeed, having regard to the life-shortening, stunting effect of many callings, it is a serious question whether a man unable otherwise to make a living should accept the responsible rôle of parent.

Does anyone really suppose that it is cheaper or more effective in the long run to so overdo the division of labour and use of mechanical process? The startling figures denoting proposed expenditure on measures to improve the national health standard are part of the cost. The money could not at present be more profitably spent. Great numbers of our fellow-men have been put through the civilisation machine to emerge as by-products for the scrap heap. The enormous cost of tinkering where possible will cause the least reflective person to realise the need of radical amendment of the system, and make his effort to bring about conditions favourable to prevention.

It is absolutely essential that each unit should at least have the opportunity of varied and balanced mental and physical activity. It is a lamentable fact that very many people are actually alienated from the natural elements, and positively shrink from a light shower or moderately boisterous breeze.

It seems reasonable to think that no part of the proposed expenditure could be better applied than in careful experiment, in some garden city, to simplify living to approximate more nearly to natural conditions. Clean blood, good digestion, sound lungs, un-"spec"'d eyesight, and unpaid-for teeth are, perhaps, as valuable national assets as flourishing trade and industrial prosperity.—Yours, etc.,

Contemplator.

[There is, undoubtedly, much in the contention which our correspondent puts forward. At the same time, it is questionable whether Britain would support anything like the population now existing without that exploitation of mineral wealth by means of "specialised" industry, which he regards as the root source of the unsatisfactory state of the national health. He would argue, we suppose, that excess of population is no desideratum, and that there is ample room for the excess to live the simple life in our extensive and only half-filled Colonies; but this overlooks the fact that we have been a Colonial nation for centuries, and that presumably our surplus population does not emigrate because it does not want to. It also neglects the consideration that only by specialised industrial activity can a modern nation maintain its freedom (and that of its colonies) by force of arms against greedy and unscrupulous aggressors of the Prussian type. When the wolf lies down with the lamb, and the German with the Conscientious Objector, possibly nations will be able to disregard the important question of self-preservation; but the lessons of the last few years, even of the last few days, do not encourage the belief that this time has yet arrived.—Ed. The Hospital.]

